# Identification of conserved canonical marker genes in human and mouse adrenal glands using Visium spatial transcriptomics

**DOI:** 10.1007/s00418-025-02446-6

**Published:** 2025-12-24

**Authors:** Małgorzata Blatkiewicz, Marta Szyszka, Szymon Hryhorowicz, Joanna Suszyńska-Zajczyk, Andrea Porzionato, Adam Plewiński, Ludwik K. Malendowicz, Marcin Rucinski

**Affiliations:** 1 Department of Histology and Embryology, Spatial Transcriptomics Laboratory, Poznan University of Medical Sciences, Swiecickiego 6, 60-781 Poznan, Poland; 2 Department of Histology and Embryology, Poznan University of Medical Sciences, Swiecickiego 6, 60-781 Poznan, Poland; 3 Polish Academy of Sciences, Institute of Human Genetics, Strzeszynska 32, 60-479 Poznan, Poland; 4 Experimental Medicine Centre, Poznan University of Medical Sciences, Rokietnicka 8, 60-806 Poznań, Poland; 5 Department of Biochemistry and Biotechnology, Poznan University of Life Sciences, Dojazd 11, 60-632 Poznan, Poland; 6 Institute of Human Anatomy - Department of Neuroscience, University of Padova, Via Aristide Gabelli 65, 35121 Padua, Italy; 7 Centre for Advanced Technologies, Adam Mickiewicz University, Uniwersytet Poznanski 10 Street, 61-614, Poznan, Poland; 8 Department of Bioinformatics and Computational Biology, Poznan University of Medical Sciences, Swiecickiego 6, 60-781 Poznan, Poland

**Keywords:** Adrenal gland architecture, Spatial transcriptomics, Canonical marker genes

## Abstract

The adrenal glands are essential endocrine organs whose cortex and medulla maintain systemic homeostasis and mediate stress responses via steroid hormone and catecholamine secretion. Despite anatomical and functional similarities between human and mouse adrenal glands, notable species-specific differences exist. Here, we leveraged spatial transcriptomics (10× Genomics Visium) to comprehensively map gene expression in adult human and mouse adrenal glands, aiming to identify canonical marker genes conserved across species. The analysis was based on a 31-year-old female human sample (GEO dataset) and four 10-week-old male CD-1 mice. Human adrenal sections were processed using optimal cutting temperature (OCT) embedding, whereas mouse adrenal sections were processed as formalin-fixed paraffin-embedded (FFPE) samples, highlighting differences in sample preparation. Using unsupervised clustering of spatial gene expression data, we delineated distinct adrenal cortex and medulla zones in both species, confirming known zonation patterns. Our cross-species analysis revealed highly conserved spatial expression of key known marker genes characteristic of the adrenal cortex (e.g., *CYP11B2* for ZG, *CYP11B1* for ZF) and medullary chromaffin cells (e.g., *TH*), as well as a core set of additional marker genes previously less characterized in adrenal biology. By integrating transcriptional profiles, we generated a catalogue of conserved canonical marker genes that define adrenal zonation and function in both humans and mice. These results highlight the fundamental molecular conservation of adrenal gland organization and support the translational value of mouse models in adrenal research. Our findings provide new insights into the evolutionary preservation of adrenal function and a valuable resource for studies on adrenal physiology and disease.

**Supplementary Information:**

The online version contains supplementary material available at 10.1007/s00418-025-02446-6.

## Introduction

The adrenal glands play a key role in maintaining physiological homeostasis, mainly through the production of steroid hormones and catecholamines, which regulate metabolism, fluid balance, and stress responses. (Ehrhart-Bornstein and Bornstein 2008). In mammals, each adrenal gland consists of an outer cortex and an inner medulla, which have distinct embryological origins and functions. The adrenal cortex is further organized into concentric zones—the zona glomerulosa (ZG), zona fasciculata (ZF), and zona reticularis (ZR) in adult humans—that synthesize various steroid hormones: mineralocorticoids, glucocorticoids, and adrenal androgens, respectively (Pihlajoki et al. 2015). The cortical zones exhibit significant differences in terms of cellular architecture and organizational patterns. The structure of the ZG, which primarily produces mineralocorticoids such as aldosterone, which regulate electrolyte and fluid balance, is highly conserved among species (Nussdorfer 1980; Vinson 2009). The ZF synthesizes glucocorticoids like cortisol in humans and corticosterone in rodents, essential for glucose metabolism and the stress response (Oakley and Cidlowski 2013). Cells in the ZF are larger and less densely packed that those in the ZG, organized in cord-like structures, which enable efficient hormone exchange between the bloodstream and steroid-producing cells. The ZR produces adrenal androgens that contribute to sexual development and function (Huang and Kang 2019; Rege and Rainey 2012). In humans, the ZR is a well-defined innermost layer of the adrenal cortex that becomes prominent after adrenarche and is responsible for the production of dehydroepiandrosterone (DHEA) and its sulphate (DHEAS), which serve as precursors for sex hormone synthesis (Nguyen and Conley 2008). The development and function of the human ZR are crucial for the onset of puberty and have implications for disorders such as congenital adrenal hyperplasia and adrenal insufficiency (Auchus and Rainey 2004). In contrast, mice lack a distinct ZR equivalent to that in humans. Instead, they possess a transient cortical layer known as the X-zone (ZX), located between the ZF and the medulla (Beuschlein et al. 2002; Hershkovitz et al. 2007). The X-zone is a temporary, fetal-origin region and persists until puberty in male mice, after which it regresses and disappears, with a cell size smaller than in the ZF (Sato 1968; Zubair et al. 2008). In female mice, the X-zone persists until the first pregnancy or extends into adulthood if the mice remain nulliparous (Bielohuby et al. 2007). The regression of the X-zone is thought to be influenced by gonadal hormones, but its exact function remains unclear (O'Shaughnessy et al. 2003). Some studies suggest that the X-zone may produce steroid hormones, but its role does not directly parallel the androgen-producing function of the human ZR (Keeney and Mason 1992). While ZG and ZF produce the same hormones as humans in rodents, the importance of the X-zone is still not fully understood.

The distinct division of the adrenal cortex and medulla into individual zones is not only related to the distinct cell populations and functional organization characteristic of both species. The presence of distinct morphological zones within the adrenal cortex, despite the absence of physical boundaries, suggests the involvement of precise molecular mechanisms that govern zonal identity.

The adrenal cortex displays a proliferative capacity, known as centripetal differentiation, where stem or progenitor cells at the adrenal capsule edge give rise to ZG cells, which then migrate inward and trans-differentiate into ZF and subsequently ZR cells (Fu et al. 2023). This phenomenon is most often induced by natural factors, such as a response to apoptosis or cell field renewal, but can also be induced by stress. The regulation of this process occurs in both the endocrine and paracrine pathways. However, due to the complex structure and characteristics of the individual layers of the adrenal cortex, cells undergoing differentiation experience not only morphological changes but also molecular changes that enable them to perform a specific function. This continuous cell transformation has been observed in both human and mouse models (Fu et al. 2023; Blatkiewicz et al. 2025). Furthermore, the adrenal glands are surrounded by an abundance of adipose tissue, including both brown adipose tissue (BAT) and white adipose tissue (WAT). These tissues may also play a substantial role in regulating adrenal function.

The identification of canonical marker genes in both murine and human models provides a fundamental basis for translational research concerning adrenal functioning and development. The identification of these genes, which exhibit stable and specific expression patterns in specific adrenal zones, facilitates not only precise histological tissue characterization but also the comparison of their activity in physiological and pathological conditions. Despite the anatomical and functional differences in the ZR/ZX, the adrenal glands of mice and humans share remarkable similarities in terms of overall structure, zonation (ZG and ZF), and hormonal functions (Mitchell et al. 2008). Both species exhibit conserved mechanisms in steroidogenesis pathways and adrenal medulla functions, making mice an excellent model for studying many aspects of human adrenal biology (Basham et al. 2016). By identifying conserved canonical marker genes across species, researchers can enhance the validation of mouse models and ensure the translational relevance of animal studies to human health. This, in turn, improves our understanding of adrenal biology and facilitates the translation of preclinical findings into clinical applications (Finco et al. 2019; Perlman 2016; Yu et al. 2015).

In this study, we employed Visium spatial transcriptomics to perform a comprehensive analysis of gene expression in human and mouse adrenal glands. We aimed to identify conserved canonical marker genes across species, focusing on the distinct zones of the adrenal cortex and medulla. By integrating spatial gene expression data and performing cross-species comparisons, we sought to elucidate the molecular underpinnings of adrenal gland zonation and highlight functional similarities between humans and mice. Our findings contribute to the foundational knowledge of adrenal gland biology and have potential implications for the development of targeted therapies for adrenal disorders.

## Materials and methods

### Mouse adrenal gland processing

All experiments involving mice (*Mus musculus*) were conducted in compliance with Directive 2010/63/EU and the Polish Act on the Protection of Animals Used for Scientific or Educational Purposes. The protocol was approved by the Local Ethics Committee for Animal Studies in Poznan, Poland (protocol No. 27/2023, April 17, 2023). Animal welfare was regularly monitored by assessing activity, behaviour, food and water intake, and the condition of fur and stool. Four sexually mature male CD-1^®^ IGS mice (10 weeks old, ~37 g) were used. Animals were bred and maintained at the Centre for Advanced Technology, Adam Mickiewicz University (Poznan), under specific-pathogen-free (SPF) conditions (21–23 °C, 55–60% humidity, 12/12 h light/dark cycle, ad libitum access to chow and water). Mice were euthanized by decapitation; both adrenal glands were immediately collected and fixed in 10% buffered formalin. Further details of tissue processing are provided in Blatkiewicz and Hryhorowicz (2025). For spatial transcriptomics, one adrenal gland from each mouse was selected. Regions of interest were determined based on histology (expert review), orientation (haematoxylin and eosin [H&E] staining), and RNA quality (DV200 > 50% by Agilent 2100 Bioanalyzer, Agilent Technologies, Santa Clara, CA, USA). Formalin-fixed paraffin-embedded (FFPE) blocks were sectioned at 5 μm. RNA was isolated with the RNeasy FFPE kit (73504, Qiagen, Hilden Germany). Spatial gene expression profiling was performed using the 10× Genomics Visium CytAssist (Firmware v2.3, 10× Genomics Visium CytAssist, 10× Genomics, Pleasanton, CA, USA) platform (FFPE protocol). Each of the four adrenal sections was mounted on SuperFrost™ Plus (12-550-15, Thermo Fisher Scientific, Waltham, MA, USA) slides, dried, and processed for Hematoxyline (S3309, Agilent Technologies, Santa Clara, CA, USA) and Eosine Y-solution, alcoholic (HT110116, Millipore Sigma, Darmstadt, Germany) staining and imaging (Grundium Ocus 40, Grundium Ltd., Tampere, Finland). Following decrosslinking and probe hybridization, sections were processed using the CytAssist instrument for permeabilization and RNase treatment. Complementary DNA (cDNA) synthesis and library preparation followed, and libraries were pooled and sequenced (final concentration: 650 pM; 20 μl) using an Illumina NextSeq 2000 (Illumina, Inc., San Diego, CA, USA) (P1 flow cell). Sequencing was performed with paired-end 100 × 100 base pair (bp) reads, which provides read length and depth sufficient for 10× Genomics Visium FFPE libraries. Histological images of mouse adrenal gland sections were acquired using a Grundium Ocus 40 digital slide scanner (Grundium Ltd., Tampere, Finland) equipped with a 40× objective lens (0.50, plan apochromat correction). The scanner is integrated with a CMOS colour camera (12 Mpixels). Image acquisition was performed with Grundium Ocus software v2.5, and output images were stored as high-resolution TIFF files. Images were recorded with a spatial resolution of 0.25 μm per pixel (x–y plane), and the scanned sections had a thickness of 5 μm (z-axis). Image bit depth was 24-bit RGB, and automatic detector gain settings were applied.

### Human adrenal data collection

Spatial expression data for human adrenal glands were obtained from the Gene Expression Omnibus (GEO) database under accession number GSE244084 (Fu et al. 2023). The following files were downloaded: GSE244084_barcodes.tsv, containing barcode sequences corresponding to individual spatial spots; GSE244084_features.tsv, listing the detected gene features; GSE244084_filtered_feature_bc_matrix.h5, compressed whole datasets for downstream analyses; GSE244084_tissue_images.jpg, comprising high-resolution tissue images.

### Data preprocessing and quality control

All spatial transcriptomics data were processed and analysed in R (v4.2.0) using the Seurat package (v4.0.5) (Satija et al. 2015; Team 2023), with additional visualization support from ggplot2 and patchwork (Wickham 2016; Lin Pedersen 2024).

For the human samples, spatial gene expression data were loaded using Seurat’s Load10X_Spatial function, specifying the filtered_feature_bc_matrix.h5 file. Initial quality control involved filtering spatial spots based on tissue presence as indicated in the tissue_positions_list.csv file. Gene expression counts were further filtered to include only genes expressed in at least 1% of the filtered spots. Gene identifiers were standardized to HGNC symbols using the Updated_HGNC_Symbols function to enable direct cross-species comparisons.

Mouse adrenal data underwent an analogous workflow. Orthologous gene mapping between mouse and human was performed using the Orthology.eg.db package (Carlson 2022). Mouse gene symbols were converted to their corresponding human orthologs to enable integrated analysis across species. Any resulting duplicate human gene symbols from ortholog mapping were appropriately labelled to maintain unique identifiers.

Gene expression normalization (to account for differences in sequencing depth) was performed with Seurat's NormalizeData function. Highly variable features were identified using Find Variable Features, focusing on genes exhibiting significant expression variability across spatial spots. All data were scaled with Scale Data to standardize gene expression values and minimize technical variability prior to integration and downstream analyses.

### Data integration and cross-species comparison

To enable cross-species comparisons and correct for batch effects, the spatial transcriptomics datasets from human and mouse adrenal glands were integrated using the Seurat package. Highly variable features common to both datasets were identified with the Select Integration Features function. Integration anchors were then determined using Find Integration Anchors, and the datasets were merged into a single integrated object via Integrate Data. This resulted in a unified assay that served as the basis for all downstream analyses.

Dimensionality reduction was performed using principal component analysis (PCA), with the optimal number of principal components determined by the jackstraw resampling method. Clustering of spatial spots was accomplished with the K-nearest neighbors (KNN) algorithm and Jaccard similarity, as implemented in Seurat’s FindNeighbors function. Clusters were identified with the FindClusters function, where various resolution parameters were tested. The most biologically relevant and stable clustering solution was selected based on visual inspection of Uniform Manifold Approximation and Projection (UMAP) plots and consistency of cluster markers across resolutions. UMAP was used for visualization of the integrated dataset, enabling clear delineation of the spatial distribution and relationships between cellular populations. The spatial mapping of identified clusters onto histological tissue images was achieved using Seurat’s SpatialDimPlot function.

### Identification of conserved marker genes across clusters

To identify canonical marker genes conserved between clusters from human and mouse adrenal glands, the FindConservedMarkers function from Seurat was employed. This approach involved performing differential gene expression analysis separately within each cluster and subsequently integrating the *p*-values using a meta-analysis method provided by the MetaDE package (Ma et al. 2017). Genes were considered conserved markers if they met the following criteria in both species: a log_2_ fold change greater than 1, and expression in at least 10% of cells within the identified clusters (pct > 0.1). After identifying conserved marker genes, additional processing was conducted to rank these markers based on their significance and expression levels. Specifically, the sum of the percentages of cells in the analysed cluster expressing each gene in both mouse and human samples (Mouse_pct.1 and Human_pct.1) was calculated and stored in the sum column. The average log_2_ fold change across both species was computed using the row Means function and stored in the sum Log column. A ranking score (rang) was then derived by multiplying the negative logarithm (base 2) of the minimum adjusted *p*-value (minimump_p_val) with the product of the percentage sum and the average log_2_ fold change. Finally, the marker genes were sorted in descending order based on their rank scores to prioritize the most significant conserved markers. For the top conserved marker genes, spatial feature plots were generated using Seurat's SpatialFeaturePlot function to illustrate their spatial distribution within both human and mouse adrenal gland samples. Additionally, a dot plot was created using Seurat's DotPlot function to display the expression levels of the top 40 conserved marker genes across different clusters, where applicable. The plots were customized with specific colour scales and point sizes to enhance clarity and interpretability. The raw data identifying conserved marker adrenal genes were also saved to an Excel file and included in the supplementary materials.

### Interactive atlas of canonical marker genes in human and mouse adrenal glands

To support interactive analysis and visualization of conserved canonical marker genes across human and mouse adrenal glands, we developed a Shiny-based web application (Chang et al. 2012). The platform integrates spatial transcriptomic datasets from both species, enabling users to dynamically explore the spatial distribution of selected marker genes within human and mouse adrenal tissue sections. The user interface was constructed with Shiny and mini UI (Cheng 2018). Data visualization was powered by ggplot2, with custom colour schemes generated via RColorBrewer (Neuwirth 2022) and colorRampPalette. Users are able to search and select any gene identified in the analysis as a canonical marker, and immediately visualize its spatial expression profile in the context of both human and mouse adrenal anatomy. The application is available at: https://adrenal-spatall-transcriptomic.shinyapps.io/canonicalmarkers/.

### Functional annotation using DAVID

To understand the biological significance of the conserved marker genes, functional annotation was performed using the DAVID (Database for Annotation, Visualization, and Integrated Discovery) web service (Dennis et al. 2003). The following steps were undertaken: Human orthologs of mouse genes were identified using the biomaRt package to ensure accurate cross-species functional analysis (Durinck et al. 2009). Gene Ontology (GO) and Kyoto Encyclopedia of Genes and Genomes (KEGG) pathway enrichment analyses were performed using the DAVID web service accessed via the RDAVIDWebService R library (Fresno and Fernandez 2013). For each annotation category (GOTERM_BP_FAT, GOTERM_MF_FAT, GOTERM_CC_FAT, KEGG_PATHWAY), the top 10 enriched terms were identified and visualized using the ggplot2 library, displaying the enriched GO terms and KEGG pathways along with their respective gene counts.

### Conservation specificity analysis

The specificity of cross-species transcriptional conservation between human zona reticularis (ZR) and mouse brown adipose tissue (BAT) was evaluated. A ZR–BAT module score was computed on mouse spatial transcriptomics data using the “AddModuleScore” function from Seurat (v4.0.5). The module was defined by an 18-gene signature derived from the integrated ZR(h)–BAT(m) cluster, and the resulting scores were calculated for each mouse spatial spot and compared across the identified clusters (ZG, ZF, BAT, medulla, CT/WAT).

The discriminatory performance of this score was assessed by receiver operating characteristic (ROC) analysis with the pROC package (v1.18.5), treating ZR(h)/BAT(m) as the positive class and all other clusters as the negative class. The area under the ROC curve (AUC) was used as a measure of separability.

Differential expression analysis was carried out for human and mouse datasets using the FindMarkers function in Seurat. For humans, the ZR(h)–BAT(m) cluster was contrasted with the remaining adrenal clusters, and for mice, each cluster was compared with all others. Average log_2_ fold change (log_2_FC) values were obtained and used to calculate Spearman’s rank correlation coefficients (ρ) between the expression profiles of human ZR and each mouse cluster across the 18 signature genes. Specificity was defined as the difference (Δρ) between the correlation observed for ZR(h)–BAT(m) and the next highest correlation among the remaining mouse clusters.

To determine whether the observed correlation between ZR(h) and BAT(m) could occur by chance, a permutation test was performed. In each of 5000 iterations, 18 random genes were sampled from the background of expressed genes, and correlations between ZR(h) and BAT(m) log_2_FC values were recalculated. The resulting distribution was used to compute an empirical *p*-value, representing the proportion of permuted correlations equal to or greater than the observed value.

## Results

**Fig. 1:**
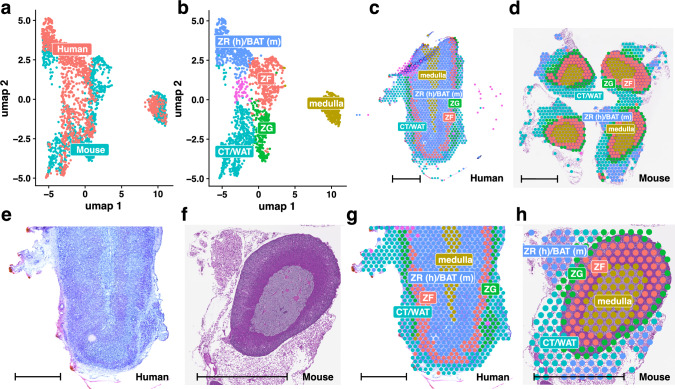
Uniform Manifold Approximation and Projection (UMAP) data reduction and cluster identification of human and mouse adrenal glands. **A** UMAP visualization of integrated and normalized spatial expression data from human (red) and mouse (turquoise) adrenal glands. **B** UMAP data reduction displaying distinct clusters identified and marked with different colours. **C**, **D** UMAP clusters depicted as color-coded dots overlaid on spatial transcriptomic adrenal slides of human (**C**) and mouse (**D**). **E**, **F** Enlarged sections of histological slides of human (**E**) and mouse (**F**) adrenal glands stained with hematoxylin and eosin (H&E). **G**, **H** Identified cell types assigned to the respective clusters displayed on the enlarged sections of the human (**G**) and mouse (**H**) adrenal glands. Sample metadata: human donor—female, 31 years old; mouse donor—male CD-1, 10 weeks old. The scale bar represents 2 mm in the human sample (1C), 1 mm in the mouse tissue (1D) and human samples (1E, G), and 500 µm in the mouse tissue samples (1F, H)

After data preprocessing and primary filtering, the spatial gene expression datasets for the adrenal glands contained 21,190 genes in humans and 19,250 genes in mice. The spatial transcriptomic data included 1073 spots from the histological section of the human adrenal gland and 992 spots from the mouse adrenal gland sections. To integrate the human and mouse datasets and correct for batch effects, we used Seurat's integration workflow. The SelectIntegrationFeatures function identified 3054 common highly variable genes across both species, serving as anchors for data integration. Next, we performed PCA on the integrated dataset to reduce dimensionality, retaining the first 30 principal components. The UMAP was then applied using these components for subsequent analyses and visualization. Figure 1A presents the UMAP dimensionality reduction of the integrated dataset, partitioned into spots from human and mouse adrenal glands. The spots are located in similar positions on the plot, suggesting successful data integration and elimination of batch effects. Next, we determined the number of clusters for the integrated dataset by using different resolution parameters ranging from 1 to 0.1. Visual inspection of the identified clusters indicated that a resolution of 0.4 provided optimal separation, and this resolution was selected for further analyses and data visualization. The results are presented in Fig. 1B, which shows the UMAP dimensionality reduction at a resolution of 0.4, displaying distinct clusters identified and marked with different colours. These clusters are spatially mapped onto the adrenal gland slides of humans (Fig. 1C) and mice (Fig. 1D). As a result of the analysis, five clusters were identified corresponding to (1) ZG, (2) ZF, (3) ZR in humans, which corresponds to BAT in mice (ZR(h)/BAT(m)), (4) the adrenal medulla, and (5) a cluster comprising connective tissue and white adipose tissue (CT/WAT). The UMAP visualization also revealed an additional cluster located within the overlaid human adrenal tissue (shown in pink). This cluster was considered an artefact and was excluded from further analyses. Notably, the cluster assigned to the adrenal medulla was the most distant from the other clusters, appearing separately in the UMAP visualization at a considerable distance from the other clusters. To improve visualization of the spatial clustering of expression profiles in human and mouse adrenal glands, higher-magnification images of the H&E-stained sections are presented in Fig. 1E and F, along with overlays of spots assigned to the corresponding cluster colours in Fig. 1G and H. Notably, in human adrenal glands, expression was strictly limited to the ZF, whereas in mouse adrenal glands, the applied clustering classified the entire adrenal cortex below the zona glomerulosa as zona fasciculata.

Canonical marker genes conserved between the human and mouse adrenal glands were identified for the selected clusters using the FindConservedMarkers function from the Seurat library. To highlight the most characteristic genes conserved between humans and mice and expressed in the same clusters, an additional selection was performed. Only genes with a log_2_ fold change greater than 1 and expression in at least 10% of cells within the identified clusters (pct.1 > 0.1) in both species were included in the final list. For the selected genes, a ranking score (rank) was calculated, allowing them to be ordered from the most significant in each cluster. The comprehensive list of the selected canonical marker genes is presented in the supplementary material (Suppl. 1).

**Fig. 2:**
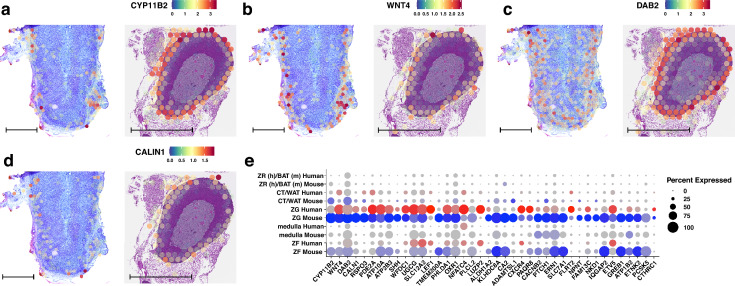
Spatial expression profiles of conserved genes corresponding to the zona glomerulosa cluster of the adrenal gland in human (left panel) and mouse (right panel). The following genes are shown: **A** *CYP11B2*, **B** *WNT4*, **C** *DAB2*, **D** *CALIN1*. **E** Dot plots of conserved genes from the zona glomerulosa cluster of the adrenal gland. Genes that passed the established cut-off criteria for conserved gene selection in the ZG cluster are displayed on the x-axis. The size of the dots corresponds to the percentage of spots assigned to the cluster where the gene was expressed. The colour intensity is inversely proportional to the *p*-value; more intense red or blue colours indicate lower *p*-values (higher statistical significance). The scale bar is 1 mm

Using the described approach, we identified 62 canonical marker genes that are conserved between the zona glomerulosa of the human and mouse adrenal glands. The spatial expression patterns of the four highest-ranking genes are presented in Fig. 2. These genes, conserved in both mice and humans, include *CYP11B2* (cytochrome P450 family 11 subfamily B member 2, aldosterone synthase), *WNT4* (Wnt family member 4), *DAB2* (disabled homolog 2), and *CALN1* (calneuron 1). The expression of the *CYP11B2* in humans within the ZG is discontinuous and occurs in clusters of cells (expressed in 72.3% of human ZG cells; Fig. 2A). In contrast, in the mouse adrenal glands, the expression of *CYP11B2* is continuous and present throughout the entire ZG (expressed in 97.1% of mouse ZG cells). The expression of the *WNT4* and *DAB2* is more continuous in the ZG of humans, with expression observed in 93.1% and 98.9% of human ZG cells, respectively (Fig. 2B, C). Similarly, in the mouse ZG, *WNT4*, and *DAB2* are expressed in 89.4% and 96.8% of ZG cells, respectively. Additionally, the expression of the *DAB2* gene is diffuse, encompassing the deeper regions of the adrenal cortex in both human and mouse adrenal glands (Fig. 2C). The expression of the *CALN1* was similar to that of the *CYP11B2*, occurring in clusters of cells (expressed in 60.6% of human ZG cells) and encompassing 88% of the mouse ZG (Fig. 2D).

**Fig. 3:**
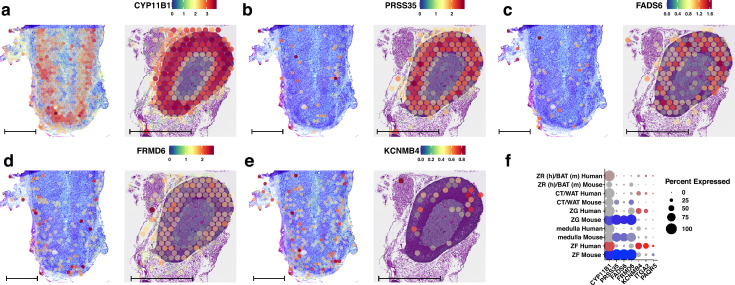
Spatial expression profiles of conserved genes associated with the zona fasciculata cluster of the adrenal gland in humans (left panel) and mice (right panel). The following genes are shown: **A** *CYP11B1*, **B** *PRSS35*, **C** *FADS6*, **D** *FRMD6*, **E** *KCNMB4*. **F** Dot plots of conserved genes from the zona fasciculata cluster of the adrenal gland. Genes that passed the established cut-off criteria for conserved gene selection in the ZF cluster are displayed on the x-axis. The size of the dots corresponds to the percentage of spots assigned to the cluster where the gene was expressed. The colour intensity is inversely proportional to the *p*-value; more intense red or blue colours indicate lower *p*-values (higher statistical significance). The scale bar is 1 mm

We identified only seven canonical conserved marker genes for the ZF in both human and mouse adrenal glands. The spatial expression patterns of the five highest-ranking genes are presented in Fig. 3. These conserved genes in both species include *CYP11B1* (cytochrome P450 family 11 subfamily B member 1), *PRSS35* (serine protease 35), FADS6 (fatty acid desaturase 6), *FRMD6* (FERM domain-containing 6), and *KCNMB4* (potassium calcium-activated channel subfamily M regulatory beta subunit 4). The highest level of *CYP11B1* gene expression was observed in the ZF. *CYP11B1* expression was also elevated in the human ZR. In mouse adrenal glands, *CYP11B1* expression was detected throughout the entire adrenal cortex. Notably, in both human and mouse adrenal glands, *CYP11B1* was expressed in 100% of the spots within clusters classified as ZF.

**Fig. 4:**
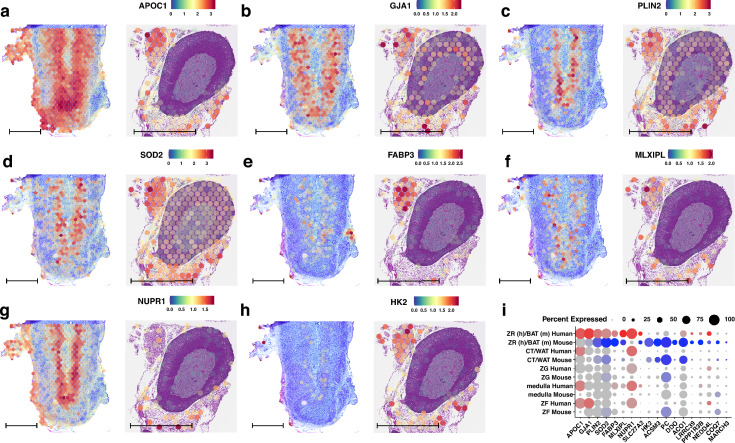
Spatial expression profiles of conserved genes associated with the zona reticularis cluster of the human adrenal gland (left panel) and with brown adipose tissue in a mouse histological section (right panel). The following genes are shown: **A** *APOC1*, **B** *GJA1*, **C** *PLIN2*, **D** *SOD2*, **E** *FABP3*, **F** *MLXIPL*, **G** *NUPR1*, **H** *HK*. **I** Dot plots of conserved genes from the zona fasciculata cluster of the human adrenal gland (left panel) and brown adipose tissue in a mouse histological section. Genes that passed the established cut-off criteria for conserved gene selection in the ZF (human) and BAT (mouse) clusters are displayed on the x-axis. The size of the dots corresponds to the percentage of spots assigned to the cluster where the gene was expressed. The colour intensity is inversely proportional to the *p*-value; more intense red or blue colours indicate lower *p*-values (higher statistical significance). The scale bar is 1 mm

We identified a set of 18 canonical marker genes conserved between the human ZR and the BAT present in the mouse histological sections. Due to anatomical differences between species, the mouse adrenal gland lacks a distinct ZR; instead, the corresponding cluster aligns with BAT in the mouse sample. The spatial expression patterns of the highest-ranking conserved genes are presented in Fig. 4. These genes include *APOC1* (apolipoprotein C1), which showed high expression levels, being present in 100% of spots within the human ZR cluster and 95.9% of spots in the mouse BAT cluster (Fig. 4A). It had the highest-ranking score (rang = 959.6), indicating strong conservation and significance across species. *GJA1* (gap junction alpha-1) was expressed in 98.8% of human ZR spots and 79.4% of mouse BAT spots (Fig. 4B), with a high-ranking score of 691.6. *PLIN2* (perilipin 2) was detected in 86.3% of human ZR spots and 94.8% of mouse BAT spots (Fig. 4C), with a ranking score of 600.8. *SOD2* (superoxide dismutase 2) showed nearly ubiquitous expression, present in 93.3% of human ZR spots and 99% of mouse BAT spots (Fig. 4D). *FABP3* (fatty-acid-binding protein 3) was expressed in 60.4% of human ZR spots and 77.3% of mouse BAT spots (Fig. 4E). This gene is involved in fatty acid transport, supporting the notion of metabolic similarities. *MLXIPL* (MLX interacting protein-like) expression was observed in 67.7% of human ZR spots and 52.6% of mouse BAT spots (Fig. 4F), with a ranking score of 443.5. This gene is associated with glucose metabolism and lipid biosynthesis. *NUPR1* (nuclear protein 1) was highly expressed in the human ZR cluster (99.5% of spots) but less so in the mouse BAT cluster (30.9% of spots) (Fig. 4G). Despite the lower expression in mice, it had a significant ranking (rang = 350.7). *HK2* (hexokinase 2) showed moderate expression in the human ZR cluster (10.9% of spots) and higher expression in the mouse BAT cluster (84.5% of spots) (Fig. 4H). Its involvement in glycolysis highlights shared metabolic pathways.

**Fig. 5:**
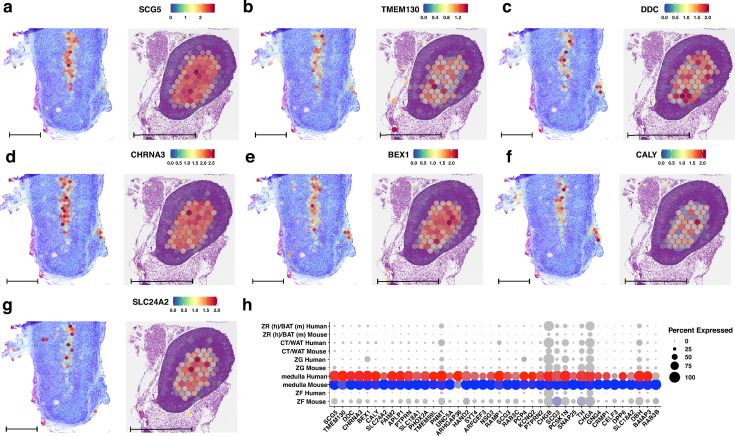
Spatial expression profiles of conserved genes associated with the adrenal medulla cluster in humans (left panel) and mice (right panel) are shown. The following genes are depicted: **A** *SCG5*, **B** *TMEM130*, **C** *DDC*, **D** *CHRNA3*, **E** *BEX1*, **F** *CALY*, and **G** *SLC24A2*. **H** Dot plots of conserved genes from the adrenal medulla cluster. The 40 genes that met the established cut-off criteria for conserved gene selection in the medulla cluster are displayed on the x-axis (additional genes are provided in the supplementary material). The size of the dots corresponds to the percentage of spots assigned to the cluster where the gene was expressed. The colour intensity is inversely proportional to the *p*-value; more intense red or blue colours indicate lower *p*-values (higher statistical significance). The scale bar is 1 mm

Using the same approach, we identified 697 canonical marker genes conserved between the adrenal medulla of human and mouse adrenal glands. The spatial expression patterns of the highest-ranking genes are presented in Fig. 5. These conserved genes include *SCG5* (secretogranin V), *TMEM130* (transmembrane protein 130), *DDC* (dopa decarboxylase), *CHRNA3* (cholinergic receptor nicotinic alpha 3 subunit), *BEX1* (brain expressed X-linked 1), *CALY* (neuron-specific vesicular protein calcyon), and *SLC24A2* (solute carrier family 24 member 2). *SCG5* emerged as the top-ranked conserved marker gene for the adrenal medulla, with expression observed in 100% of the spots assigned to this cluster in both humans and mice (Fig. 5A). The average log_2_ fold change for *SCG5* was 4.88 in mice and 5.38 in humans, indicating a substantial upregulation in the adrenal medulla compared to other clusters. *TMEM130* showed high expression levels in the adrenal medulla, with average log_2_ fold changes of 4.69 in mice and 4.76 in humans (Fig. 5B). It was expressed in approximately 97% of mouse adrenal medulla spots and 98% of human adrenal medulla spots. *DDC*, encoding dopa decarboxylase, was also prominently expressed in the adrenal medulla cluster (Fig. 5C). It exhibited average log_2_ fold changes of 5.00 in mice and 4.52 in humans, with expression in 99.5% of mouse and 90% of human adrenal medulla spots. *DDC* is crucial for catecholamine biosynthesis, underscoring its significance in adrenal medulla function where catecholamines like adrenaline and noradrenaline are produced. *CHRNA3* had high expression in the adrenal medulla, with average log_2_ fold changes of 5.28 in mice and 4.51 in humans (Fig. 5D). It was expressed in 100% of mouse adrenal medulla spots and 97.5% of human spots. *CHRNA3* encodes a subunit of nicotinic acetylcholine receptors which are important for synaptic transmission in the adrenal medulla. *BEX1* and *CALY* were also among the top conserved markers (Fig. 5E and F). *BEX1* showed average log_2_ fold changes of 5.21 in mice and 3.92 in humans, with expression in nearly all adrenal medulla spots in both species. *CALY* had average log_2_ fold changes of 5.10 in mice and 4.14 in humans, with expression in over 94% of mouse and 90% of human adrenal medulla spots. *SLC24A2* was another significant conserved marker (Fig. 5G), with average log_2_ fold changes of 5.11 in mice and 4.80 in humans. It was expressed in 98.9% of mouse and 69.2% of human adrenal medulla spots. *SLC24A2* encodes a sodium/potassium/calcium exchanger, which may be involved in calcium signalling pathways within chromaffin cells.

**Fig. 6:**
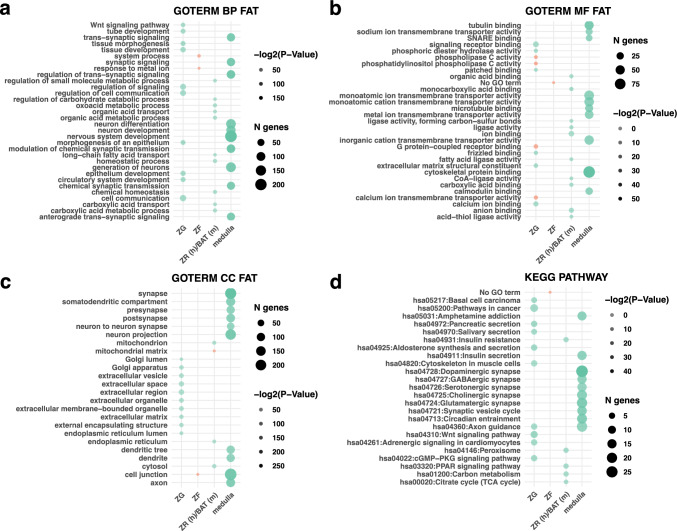
Ontology term enrichment analysis for human and mouse conserved genes from ZG, ZF, ZR (human)/BAT (mouse), and medulla. Bubble plots present the 10 ontology terms (if detected) with the lowest *p*-values for each cluster. The size of each bubble indicates the number of genes involved in regulating the process, and the transparency is negatively correlated with −log2 (*p*-value). Statistically significantly enriched terms are highlighted in turquoise, while terms with *p*-values above 0.05 are displayed in red. The analysis was performed using four reference databases from the DAVID repository: **A** GO Biological Process (BP), **B** GO Molecular Function (MF), **C** GO Cellular Component (CC), and **D** KEGG Signalling Pathway

To elucidate the biological functions and pathways associated with the conserved marker genes identified in each adrenal gland cluster, we performed GO and KEGG pathway enrichment analyses using the DAVID web service. Conserved markers of each component of human and mouse adrenal glands were included in the analysis. The top 10 enriched GO terms for each cluster were identified and visualized using bubble plots (Fig. 6), where the size of each bubble represents the number of genes associated with a term, and the colour reflects the statistical significance.

For the ZG cluster, GO Biological Process enrichment analysis revealed significant enrichment in terms related to tissue and epithelium morphogenesis. The most significantly enriched term was “morphogenesis of an epithelium” (*p* = 3.42 × 10^−9^), involving 13 genes including *WNT4*, *LEF1* (lymphoid enhancer-binding factor 1), *SHH* (sonic hedgehog), *PTCH1* (patched 1), and *RSPO3* (R-spondin 3). This suggests a pivotal role of epithelial morphogenesis in ZG function and aldosterone synthesis. Other highly enriched GO terms included “tissue morphogenesis” (*p* = 3.65 × 10^−9^), involving 14 genes including *TNNT2* (troponin T2 cardiac type) and *CXCR4* (C-X-C motif chemokine receptor 4); “epithelium development” (*p* = 2.25 × 10^−8^), with 17 genes including *KLF5* (Krüppel-like factor 5) and *UGCG* (UDP-glucose ceramide glucosyltransferase); and “tissue development” (*p* = 1.44 × 10^−7^), involving 20 genes.

In the GO Cellular Component category, the top enriched terms were “extracellular matrix” (*p* = 8.14 × 10^−5^), involving 10 genes such as *COL4A4* (collagen type IV alpha 4 chain) and *NPNT* (nephronectin) and “external encapsulating structure” (*p* = 8.24 × 10^−5^), including the same set of genes. GO Molecular Function analysis showed enrichment in “frizzled binding” (*p* = 5.73 × 10^−3^), involving *CTHRC1* (collagen triple helix repeat-containing 1), *RSPO3*, and *WNT4*, and “extracellular matrix structural constituent” (*p* = 1.54 × 10^−2^), including *COL4A4, COL4A5* (collagen type IV alpha 5 chain), *CTHRC1*, and *NPNT*.

KEGG pathway analysis identified several significantly enriched pathways. The “Wnt signalling pathway” (*p* = 7.47 × 10^−5^) involved seven genes: *WNT4, LEF1, NFATC4* (nuclear factor of activated T-cells 4), *NKD1* (naked cuticle homolog 1), *PLCB1* (phospholipase C beta 1), *RSPO3*, and *FRZB* (frizzled-related protein). “Aldosterone synthesis and secretion” (*p* = 7.08 × 10^−4^) included *ATP1B2* (ATPase Na + /K + transporting subunit beta 2), *ATP2B3* (ATPase plasma membrane Ca\2 + transporting 3), *CYP11B2*, *PDE2A* (phosphodiesterase 2A), and *PLCB1*. “Pancreatic secretion” (*p* = 9.51 × 10^−4^) involved genes including *CA2* (carbonic anhydrase 2) and *SLC12A2* (solute carrier family 12 member 2). These results underscore the importance of Wnt signalling and aldosterone biosynthesis pathways in ZG function.

Due to the limited number of conserved marker genes identified in the ZF cluster, the GO enrichment analysis yielded no statistically significant terms. The top enriched GO Biological Process terms included “response to metal ion” (*p* = 0.091), involving *CYP11B1* and *KCNMB4*, and “system process” (*p* = 0.097), including *ITGA2* (integrin subunit alpha 2). In the GO Cellular Component category, the term “cell junction” (*p* = 0.085) was enriched, involving *FRMD6*, *ITGA2*, and *KCNMB4*. KEGG pathway enrichment analysis did not yield any results for the ZF cluster, likely due to the small number of conserved marker genes.

For the zona reticularis (human)/brown adipose tissue (mouse) cluster, GO Biological Process enrichment analysis revealed significant enrichment in metabolic processes. The top enriched terms included “carboxylic acid metabolic process” (*p* = 4.20 × 10^−4^), involving genes such as *ACO1* (aconitase 1), *ACSM3* (acyl-CoA synthetase medium-chain family member 3), *DDO* (D-aspartate oxidase), *HK2*, *PC* (pyruvate carboxylase), and *SLC27A2* (solute carrier family 27 member 2). “Oxoacid metabolic process” (*p* = 4.86 × 10^−4^) included the same set of genes, indicating a role in organic acid metabolism and energy production. Other enriched GO terms included “chemical homeostasis” (*p* = 7.84 × 10^−4^), involving genes like *FABP3* and *MLXIPL*, and “long-chain fatty acid transport” (*p* = 9.78 × 10^−4^), including *PLIN2* and *SLC27A2*.

GO Molecular Function analysis showed enrichment in “monocarboxylic acid binding” (*p* = 1.97 × 10^−3^), involving *APOC1*, *FABP3*, and *PC*, and “anion binding” (*p* = 1.05 × 10^−2^), including genes like *ACSM3, DDO, HK2*, and *SLC27A2*. “Ligase activity” (*p* = 1.11 × 10^−2^) involved *ACSM3, PC*, and *SLC27A2*. KEGG pathway analysis identified the “PPAR signalling pathway” (*p* = 7.12 × 10^−3^), involving *FABP3*, *PLIN2*, and *SLC27A2*; “peroxisome” (*p* = 8.45 × 10^−3^), including *DDO, SLC27A2*, and *SOD2*; and “insulin resistance” (*p* = 1.42 × 10^−2^), involving *MLXIPL, PPP1R3B* (protein phosphatase 1 regulatory subunit 3B), and *SLC27A2*. These results suggest a role in lipid metabolism, fatty acid oxidation, and energy homeostasis, which are characteristic functions of both the *ZR* and BAT.

We identified 697 canonical marker genes conserved between the adrenal medulla of human and mouse adrenal glands. GO Biological Process enrichment analysis revealed that the most significantly enriched term was “nervous system development” (*p* = 6.70 × 10^−46^), involving 206 genes including *ASCL1* (achaete-scute family bHLH transcription factor 1), *DRD2* (dopamine receptor D2), *GATA3* (GATA binding protein 3), *NTRK1* (neurotrophic receptor tyrosine kinase 1), and *TH* (tyrosine hydroxylase). This enrichment underscores the neuroectodermal origin of the adrenal medulla and its role in neuroendocrine functions. Other highly enriched GO terms included “modulation of chemical synaptic transmission” (*p* = 2.99 × 10^−38^), involving 88 genes including *SNAP25* (synaptosomal-associated protein 25), *SYT1* (synaptotagmin 1), *STX1A* (syntaxin 1A), and *GRIA2* (glutamate ionotropic receptor AMPA type subunit 2); “regulation of trans-synaptic signalling” (*p* = 3.50 × 10^−38^), with the same set of genes; and “synaptic signalling” (*p* = 3.13 × 10^−35^), involving 82 genes.

In the GO Cellular Component category, the top enriched term was “synapse” (*p* = 3.48 × 10^−82^), with 220 genes localized to synaptic structures. Notable genes include *BSN* (bassoon presynaptic cytomatrix protein), *PCLO* (piccolo presynaptic cytomatrix protein), and *RIMS1* (regulating synaptic membrane exocytosis 1). Other enriched terms included “cell junction” (*p* = 1.70 × 10^−67^), involving 247 genes, and “neuron projection” (*p* = 4.83 × 10^−63^), with 188 genes.

GO Molecular Function analysis revealed significant enrichment in “cytoskeletal protein binding” (*p* = 8.81 × 10^−17^), involving 92 genes including *MAP1A* (microtubule-associated protein 1A), *DNM1* (dynamin 1), and *KIF5A* (kinesin family member 5A); “tubulin binding” (*p* = 1.54 × 10^−12^), including 47 genes; and “microtubule binding” (*p* = 6.56 × 10^−9^), involving 33 genes.

KEGG pathway analysis identified “dopaminergic synapse” as the most significantly enriched pathway (*p* = 1.51 × 10^−14^), involving 27 genes including *TH*, *DDC*, *DRD2*, and *SLC18A2* (vesicular monoamine transporter 2). Other significantly enriched KEGG pathways included “glutamatergic synapse” (*p* = 1.42 × 10^−9^), involving genes like *GRIA2*, *GRIA4*, and *GRIK2*; “circadian entrainment” (*p* = 4.22 × 10^−9^), including *ADCY1* (adenylate cyclase 1) and *PER3* (period circadian regulator 3); and “synaptic vesicle cycle” (*p* = 1.13 × 10^−8^), involving genes like *SNAP25, STX1A*, and *SYT1*.

**Fig. 7:**
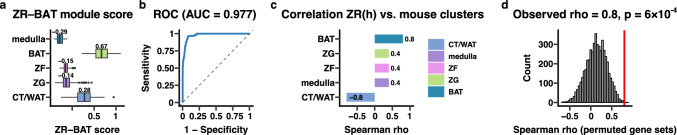
Specificity of ZR–BAT transcriptional conservation. **a** Boxplot of ZR–BAT module scores across mouse adrenal clusters. The highest median score was observed in the ZR(h)/BAT(m) cluster. **b** Receiver operating characteristic (ROC) analysis discriminating BAT from other clusters (AUC = 0.98). **c** Barplot of Spearman correlations between human ZR and mouse clusters across the 18-gene signature. ZR(h)/BAT(m) displayed the strongest correlation (ρ = 0.8), with a specificity margin of Δρ = 0.4 relative to the next best cluster. **d** Histogram of Spearman correlations from 5000 random gene sets. The observed correlation (red line) was significantly greater than expected by chance (empirical *p* = 6 × 10^−4^)

To further evaluate the specificity of transcriptional conservation between human ZR and mouse BAT, the ZR(h)–BAT(m) module score was examined across mouse adrenal clusters. The highest median score was observed in the ZR(h)–BAT(m) cluster (0.67), whereas lower median values were obtained in CT/WAT (0.28), ZG (–0.14), ZF (–0.15), and medulla (–0.29). This distribution indicated strong enrichment of the ZR–BAT signature in the ZR(h)/BAT(m) cluster relative to the other compartments (Fig. 7a). ROC analysis demonstrated excellent discrimination of BAT from other mouse clusters, with an AUC of 0.98 (Fig. 7b). At the level of differential expression, Spearman correlation of log_2_FC values between human ZR and mouse clusters across the 18 signature genes reached ρ = 0.8 for ZR(h)/BAT(m). The correlations for other clusters were considerably lower (ρ = 0.4 for ZF, ZG, and medulla; ρ = –0.8 for CT/WAT), yielding a specificity margin Δρ = 0.4 (Fig. 7c). Permutation analysis further supported this observation. When 18 random genes were sampled 5000 times, the observed correlation of 0.8 was highly unlikely to occur by chance (empirical *p* = 6 × 10^−4^; Fig. 7d). These findings provide strong evidence that the ZR–BAT transcriptional similarity is highly specific and not shared with other murine adrenal or adipose clusters.

## Discussion

The identification of canonical marker genes in the human and mouse adrenal glands is a pivotal step in comprehending the evolutionarily conserved mechanisms of differentiation and function of individual adrenal cortex zones. The genes in question are expressed in specific regions of the adrenal gland in a precise and stable manner. This enables the organ to be mapped both structurally and functionally, both under physiological conditions and in the context of pathologies. The cross-species comparison facilitated by this methodology enables not only the verification of animal models but also the assessment of the extent to which results obtained in preclinical studies in mice can be transferred to the human context. Conserved marker genes represent a significant research tool in the study of adrenal gland spatial organization, zonation plasticity monitoring, and the identification of molecular changes associated with the development of endocrine diseases. The absence of a definitive definition engenders intricacy in the interpretation of transcriptomic data and imposes limitations on the translation of experimental outcomes. It is imperative to emphasize the significance of precise characterization and comparison of canonical markers across species. This is of fundamental importance for enhancing the quality of mouse models and for designing more effective therapeutic strategies in human medicine.

Herein, we utilized Visium spatial transcriptomics (10× Genomics) to identify conserved canonical marker genes between human and mouse adrenal glands. By integrating spatial gene expression data and performing cross-species comparisons, we aimed to elucidate the molecular underpinnings of adrenal gland zonation and highlight functional similarities and differences across species. Firstly, the spatial zonation of the adrenal cortex is indicated in both humans and mice, with a focus on the preservation of homologous functional regions. In both species, zones corresponding to ZG, ZF, and ZR, or corresponding regions rich in BAT cells were identified. Furthermore, the medullary space (medulla) and the connective and white adipose tissue zone (CT/WAT), which play an important role in adrenal architecture, were distinguished. The employment of spatial transcriptomics has facilitated the precise mapping of the functional profile of the adrenal gland. This has led to the confirmation that the molecular identity of the adrenal cortex zones is well conserved across species. Consequently, this methodology can be effectively utilized to identify homologous cell populations and draws attention to the translational value of the mouse model.

In this study, we identified a set of genes that show a clear differential expression between the ZG and ZF, confirming their usefulness as canonical markers of these zones. In the ZG, genes such as *CYP11B2, WNT4, DAB2* and *CALN1* exhibited high levels of expression that were both significant and spatially restricted. *CYP11B2*, which encodes aldosterone synthase, remains the best-characterized ZG marker, a conclusion that has been confirmed by numerous previous studies (Nishimoto et al. 2010; Nanba et al. 2013). Variations in the expression patterns of *CYP11B2* between species, with a more clustered distribution in humans and continuous expression in mice, may reflect species-specific regulatory mechanisms governing aldosterone production (Bassett et al. 2004). Aldosterone plays a critical role in electrolyte and fluid balance, and its dysregulation can lead to conditions such as hyperaldosteronism (Funder 2015).

Additionally, *WNT4*, a component of the WNT/β-catenin signalling pathway, has been identified as a regulatory factor for the homeostasis and proliferation of ZG cells (Drelon et al. 2016). WNT signalling has been demonstrated to play a critical role in maintaining ZG identity by repressing zona fasciculata (ZF) fate. Constitutive PKA activity in the ZF suppresses WNT4 expression, thereby permitting glucocorticoid-producing differentiation, whereas ZG cells require active WNT signalling to sustain CYP11B2 expression (Drelon et al. 2016). Of particular significance is the finding that the aldosterone pathway (renin–angiotensin–aldosterone system) and WNT signalling are functionally interconnected. Angiotensin II has been demonstrated to upregulate WNT4, which in turn stabilizes CYP11B2 transcription, thus generating a positive feedback loop that is essential for efficient aldosterone synthesis. The molecular basis for ZG-specific function is provided by crosstalk between WNT and RAAS pathways, emphasizing the indispensability of both pathways for maintaining aldosterone production and electrolyte homeostasis.

Furthermore, the analysis of conserved ZG markers, including *CYP11B2*, *WNT4*, *DAB2* and *CALN1*, has revealed a molecular framework that safeguards ZG identity and integrates extracellular signals with steroidogenesis. *CYP11B2* is responsible for encoding aldosterone synthase, which is the terminal enzyme that catalyses the conversion of 17β-hydroxy-4-androsten-3-one (DOC) to aldosterone. The ZG-restricted, clustered pattern of *CYP11B2* in humans (in contrast to the continuous band observed in mice) underscores the species-specific regulation of aldosterone output (Gomez-Sanchez et al. 2020; Nishimoto et al. 2010). WNT4 has been demonstrated to drive canonical Wnt/β-catenin signalling, thereby promoting ZG proliferation and survival and indirect upregulation of CYP11B2. Within this regulatory network, DAB2 and CALN1 serve as additional effectors. DAB2, an adaptor protein linked to the TGF-β/Smad pathways, has been demonstrated to enhance CYP11B2 transcription and to integrate growth factor cues with steroidogenesis (Seccia et al. 2017). CALN1 modulates intracellular Ca^2+^ dynamics, a pivotal second messenger for angiotensin II- and K^+^-stimulated CYP11B2 activation. At the same time, this module also provides a mechanistic substrate for the occasional glucocorticoid-related transcription observed in ZG under conditions of zonal plasticity. Although CYP11B1 is normally confined to the ZF, sporadic expression has been documented in ZG during development, ageing, and pathology (Duparc et al. 2022). The interplay of WNT signalling, calcium-dependent pathways (CALN1), and growth-factor inputs (DAB2) may create a permissive environment that transiently enables such expression. Thus, these conserved genes not only stabilize the mineralocorticoid phenotype but also explain the limited glucocorticoid signals sporadically detected in ZG, underscoring their dual role in maintaining identity and allowing adaptive flexibility.

In the context of the ZF, increased expression of genes such as *CYP11B1, PRSS35, FADS6, FRMD6*, and *KCNMB4* was demonstrated. *CYP11B1*, encoding 11β-hydroxylase, is an essential known marker of cortisol synthesis and a recognized indicator of the activity of the ZF (Hattangady et al. 2012). A notable finding is the expression of *FADS6* and *KCNMB4* in ZF, which have not been extensively analysed in the context of the adrenal glands, suggesting their potential as novel markers or regulators of glucocorticoid secretion. It has been shown that in mouse models that suppressed expression of *FADS6*, which is the rate-limiting enzyme of polyunsaturated fatty acid (PUFA) synthesis, is related to diminished cholesterol import and corticosterone and aldosterone production and has an impact on the mitochondrial lipidome in adrenocortical cells (Witt et al. 2023). Another finding is in regard to the expression of *KCNMB4*, the β4 subunit of BK channels, which are calcium-activated potassium channels that are crucial for maintaining the membrane potential of steroidogenic cells (Barrett et al. 2021). The identification of *KCNMB4* as a potential marker specific to the ZF has the potential to provide new information on the functional differences between individual adrenal cortex zones. The limited number of conserved genes in the ZF may be attributed to species-specific differences in glucocorticoid synthesis and regulation. Mice primarily produce corticosterone, whereas humans synthesize cortisol, which may account for differential gene expression profiles and enzyme usage between species (Sandberg and Ji 2003; Payne and Hales 2004).

A noteworthy discovery was made when a comparison was drawn between ZR in the human adrenal cortex and the corresponding structures in mice (BAT). A total of 18 canonical marker genes were identified as being conserved between these regions. The present findings indicate a certain degree of molecular homology, insofar as the human ZR exhibits conserved gene expression profiles with those of the mouse BAT. Genes such as *APOC1*, *PLIN2*, *FABP3*, *GJA1*, and *SOD2* were selectively enriched in the inner cortical zones, consistent with their functional involvement in lipid transport, oxidative stress defence, and metabolic regulation (Yates et al. 2013; Rege and Rainey 2012). These genes have not been traditionally linked to adrenal zonation; nevertheless, their expression profiles exhibit a marked localization to regions corresponding to ZR in humans and BAT in mice. This phenomenon is indicative of the metabolic intensity of the regions in question, as evidenced by the presence of mitochondrial enrichment and elevated biosynthetic demands (Pignatti et al. 2017; Vega-Vasquez et al. 2024). It is important to note that genes such as *MLXIPL* and *HK2*, which are involved in carbohydrate metabolism and glycolytic control, also displayed zonally restricted expression (Cavalcante et al. 2016, Takao et al. 2021), further supporting the concept of a metabolically dynamic microenvironment within ZR. The presence of such markers underscores the dual role of ZR in both steroidogenesis and broader energy homeostasis.

To determine whether this overlap represented a general feature of adrenal or adipose tissues, the specificity of the ZR–BAT transcriptional similarity was formally assessed. The ZR(h)–BAT(m) module score was markedly enriched in the BAT cluster compared to other compartments, and ROC analysis confirmed excellent discrimination (AUC = 0.98). Correlation of differential expression profiles across the 18-gene signature was highest between human ZR and mouse BAT (ρ = 0.8), whereas lower values were obtained for the ZF, ZG, and medulla (ρ = 0.4) and for CT/WAT (ρ = –0.8). The specificity margin (Δρ = 0.4) and permutation analysis (*p* = 6 × 10^−4^) indicated that the observed correlation is unlikely to have occurred by chance and is specifically enriched in BAT.

These results lend support to the hypothesis that ZR and BAT, despite their divergent physiological roles, may share a conserved transcriptional program related to energy metabolism and cellular plasticity (Han et al. 2020; Cannon and Nedergaard 2004; Bartelt and Heeren 2014). At the same time, the data do not imply complete equivalence between these tissues. Rather, the overlap appears to reflect conserved metabolic pathways associated with lipid handling and energy regulation, which may arise from shared developmental cues or convergent adaptation. Furthermore, both ZR and BAT are under strong neuroendocrine control. ZR function in humans is modulated via ACTH signalling through the hypothalamic–pituitary–adrenal axis, while BAT in mice is responsive to adrenergic stimulation, particularly during thermogenic activation (Wankhade et al. 2016) This shared regulation suggests that, despite species-specific adaptations, the underlying mechanisms governing activation and energy use in these regions may be evolutionarily conserved. Collectively, these findings suggest a refined perspective on ZR, emphasizing its role not only as a steroidogenic compartment but also as a metabolically versatile zone exhibiting selective transcriptional parallels with BAT.

Recent single-cell and spatial-omics studies corroborate and extend our findings. An integrated single-cell and spatial transcriptomic analysis of early human adrenal development (Del Valle et al. 2023) described rapid cortical expansion, emergence of a definitive zone expressing *HOPX* and *RSPO3/LGR4*, and progressive acquisition of zona-specific steroidogenic programs that mirror the conserved markers reported above (Del Valle et al. 2023). A subsequent high-resolution single-cell atlas of the adult human adrenal (Iwahashi et al. 2024) mapped distinct cortical and medullary clusters, confirming the expression of *CYP11B2* in ZG, *CYP11B1* in ZF, and *SCG5/CHRNA3* in chromaffin cells, and uncovered additional stromal and immune populations that interact with these zones. The latest spatial-omics profiling (Altieri et al. 2024) refined the spatial topology of these cell types, showing that the conserved ZR-BAT signature (*APOC1*, *PLIN2*, *FABP3*) aligns with a metabolically active peri-cortical region adjacent to brown-fat-like tissue, and that medullary markers (*SCG5*, *TMEM130*) are tightly confined to the central chromaffin core. Together, the canonical marker dataset provides a cross-species reference that validates the zonal gene signatures uncovered by these newer human-focused single-cell and spatial studies, while the newer atlases add resolution on developmental dynamics, micro-environmental interactions, and metabolic specialization within each adrenal zone.

Next, we revealed a strong conservation of gene expression profiles in the adrenal medulla between humans and mice. We identified 697 canonical marker genes conserved in this region, many of which are associated with neuroendocrine functions, catecholamine biosynthesis, and synaptic transmission. The common, core expression of the *SCG5, TMEM130, DDC, OCHNA3, BEX1, CALY,* and *SLC24A2* genes indicates their potential role as molecular chromaffin cell markers and as an indicator of the functional identity of the adrenal core. Notably, *SCG5* emerged as the top-ranked conserved marker gene for the adrenal medulla. This gene was expressed in 100% of the spots assigned to the medulla cluster in both species and exhibited substantial upregulation compared to other clusters. This suggests that *SCG5* plays a critical role in the function of chromaffin cells in the adrenal medulla. *SCG5* acts as a molecular chaperone for prohormone convertases, facilitating the processing of neuropeptide precursors essential for catecholamine synthesis and secretion (Taupenot et al. 2003). Chromaffin cells are responsible for producing adrenaline and noradrenaline, critical hormones in the stress response, underscoring the significance of SCG5 in neuroendocrine regulation (Perry and Capaldo 2011). The conserved expression of other key genes such as *DDC*, *TH*, and *CHRNA3* further emphasizes the shared neuroendocrine functions between species. These genes are integral to catecholamine biosynthesis and cholinergic signalling, which are essential for the adrenal medulla's role in the fight-or-flight response (Eisenhofer et al. 1997; Lau et al. 1988). Furthermore, *SLC24A2*, a calcium-sodium transporter, has been demonstrated to function as an ion balancer in the medullary cells, a process which can be of significant importance for the modulation of catecholamine release. To date, the subject's expression has been predominantly characterized in the context of the nervous system. Consequently, its presence in the adrenal medulla represents a noteworthy discovery that necessitates further research (Li et al. 2020).

The use of spatial transcriptomics in this study provided a high-resolution map of gene expression within the adrenal gland, preserving the spatial context of cellular populations. This approach allowed us to discern not only the conserved genes but also their precise localization within the adrenal zones, offering insights into zone-specific functions. The integration of human and mouse data further enabled us to identify species-specific differences and similarities, which is critical for the effective translation of preclinical findings to human physiology. However, there are limitations to our study. The sample size was limited to one human and one mouse dataset, which may not capture the full variability inherent in these species. Additionally, the adrenal gland exhibits sexual dimorphism and age-related changes that were not accounted for in this analysis (Ishimoto and Jaffe 2011). Moreover, the defined spots, despite their deconvolution, do not clearly indicate expression in specific cells, due to the limitation of the available resolution. However, they provide some starting information for spatial analyses at the single cell level. Future studies incorporating larger sample sizes, including both sexes and a range of ages, would provide a more comprehensive understanding of adrenal gland gene expression profiles.

Our spatial transcriptomics analyses have revealed a highly organized, zone-specific expression of canonical genes in human and mouse adrenal glands. This confirms the behaviour of the conservative molecular program within the cortex and medulla, and identifies new, potentially translational markers characteristic of individual functional zones of this organ. The identification of markers represents the initial phase in the context of future functional research. The overarching objective of this research is to facilitate a comprehensive understanding of the mechanisms that regulate zone identity, the plasticity of steroidogenic cells, and their adaptation to both physiological and pathological conditions (Malendowicz 1994). This includes, but is not limited to, the developmental process, the response to stress, and the onset and progression of cancer.

## Supplementary Information

Below is the link to the electronic supplementary material.

Supplementary file1 Conserved canonical marker genes identified in the spatial transcriptomic analysis of human and mouse adrenal glands for clusters ZG, ZF, ZR (human)/BAT (mouse), and medulla. The dataset includes the following columns: Gene (gene symbol), Mouse_p_val (raw *p*-value in mouse), Mouse_avg_log2FC (average log_2_ fold change in mouse), Mouse_pct.1 (percentage of spots within the mouse cluster where the gene is detected), Mouse_pct.2 (percentage of spots in other mouse clusters where the gene is detected), Mouse_p_val_adj (adjusted *p*-value in mouse), Human_p_val (raw *p*-value in human), Human_avg_log2FC (average log_2_ fold change in human), Human_pct.1(percentage of spots within the human cluster where the gene is detected), Human_pct.2 (percentage of spots in other human clusters where the gene is detected), Human_p_val_adj (adjusted *p*-value in human), max_pval, minimump_p_val, and rang (ranking score) (XLSX 143 KB)

Supplementary file2 Results of Gene Ontology (GO) and KEGG pathway enrichment analyses for the specified clusters. Analysis includes the following columns: Category: The GO term category (e.g., GOTERM_BP_FAT) or KEGG pathway, Term: The specific GO term or KEGG pathway name, Count: Number of genes associated with the term, *P*-Value: Raw *p*-value of the enrichment analysis, Genes: List of gene symbols associated with the term, List. Total: Total number of genes in the input list, Pop. Hits: Number of genes associated with the term in the background population, Pop. Total: Total number of genes in the background population, Fold. Enrichment: Ratio of observed gene count to expected count (enrichment score), Group: Name of the cluster (e.g., ZG, ZF, ZR/BAT, medulla) (XLSX 23 KB)

## Data Availability

The datasets generated during and/or analysed during the current study are available in the Gene Expression Omnibus (GEO) repository, under accession number GSE283302.
